# Supplementary material for: Health insurance non-enrollment among women in Sierra Leone: A cross-sectional analysis of the 2019 Demographic and Health Survey
Source: PLoS One. 2025 Jul 31;20(7):e0323538. doi: 10.1371/journal.pone.0323538 (PMC12312921; doi:10.1371/journal.pone.0323538)
Supplement: Attachment — Submitted filename: Response to Reviewers.docx [file pone.0323538.s002.docx]

The Editor

PLOS ONE

1^st^ March 2025

Ref: PONE-D-24-25104

**Title:** Determinants of Non-enrollment on Health Insurance among Women in Sierra Leone: a cross-sectional analysis of the 2019 Sierra Leone Demographic Health Survey.

Response to Reviewers' comments

Dear Sir/Madam,

We want to express our sincere thanks for painstakingly reviewing our manuscript and providing valuable comments and suggestions. Please see our point-by-point response to the reviewers' comments and suggestions. Revisions are highlighted with track changes in the revised manuscript.

**ACADEMIC EDITOR:**

The reviewers have highlighted several issues that the authors need to carefully address. It is essential to revise the manuscript accordingly, making necessary corrections or providing a well-reasoned rebuttal for each point, as appropriate.

Response: Thank you. We have carefully addressed all reviewer comments to help strengthen our manuscript.

Reviewer #1: Abstract: Introduction: The rationale of the study is poorly explained. Need to ensure the reason for the current study.  It is important to detail why women become the population group. If there is not enough space, it is alright to add that in the main text.

Response: Health insurance enrollment is a vital component of achieving universal health coverage (UHC) and improving access to essential healthcare services. However, in Sierra Leone, enrollment remains persistently low, posing a significant public health challenge. Women aged 15–49 represent a critical population for this issue due to their unique healthcare needs, particularly related to reproductive health, pregnancy, and childcare, as well as their central role in household and community health. Despite their importance, women face significant barriers to health insurance enrollment, including financial constraints, gender inequalities, and sociocultural factors. This study examines the factors associated with non-enrollment of health insurance among women in this demographic, aiming to inform targeted interventions to improve coverage and advance UHC in Sierra Leone.
Methods:  'Multivariable binary logistic regression analysis'- use standard statistical terminology. There is no reason to invent new terms such as this. I believe you have used 'multiple logistic regression'.

Response: Multiple logistic regression analysis was used to examine the factors associated with health insurance non-enrollment among the women.
Result: Currently odds ratio is hard to understand double negative direction. If non-enrolment is the outcome of interest- try switching the reference category of each variable so it is easier to understand the message. You have done that in the second half of result.

Response: The proportion of health insurance non-enrollment among the women was 96.0%. Women with higher education were significantly less likely to be uninsured (aOR: 0.35, 95% CI: 0.18-0.64, p=0.001) compared to those with no education, indicating that higher education was protective against non-enrollment. Employment also reduced the odds of being uninsured (aOR: 0.47, 95% CI: 0.36-0.62, p<0.001) compared to unemployment. Listening to the radio less than once a week was associated with lower odds of being uninsured (aOR: 0.71, 95% CI: 0.53-0.97, p=0.032) compared to not listening at all. On the other hand, women who reported distance to a health facility as a big problem were more likely to be uninsured (aOR: 2.21, 95% CI: 1.03-4.75, p=0.041) compared to those who did not consider it a problem. Regionally, women living in the Northwestern (aOR: 0.07, 95% CI: 0.03-0.14, p<0.001) and Northern regions (aOR: 0.28, 95% CI: 0.12-0.66, p=0.004) were significantly less likely to be uninsured compared to those in the Eastern region.
Conclusion: 'Again, efforts should be made to eliminate financial barriers that prevent women from being able to afford health insurance enrolment.'- The significant variables do not explain or examine financial barrier in your study.  In general men and women both who face financial barrier are at risk of not having health insurance. However, that is not what you have investigated in this study.  Please use your current results only to recommend the interventions.

Response: Health insurance non-enrollment remains very high among women in Sierra Leone.Higher education and employment were protective against non-enrollment, underscoring the need to address socioeconomic barriers and promote education and economic empowerment to increase coverage. The association between listening to the radio and reduced odds of non-enrollment suggests that leveraging mass media campaigns could be an effective strategy to raise awareness and promote health insurance uptake. Conversely, the increased likelihood of non-enrollment among women facing distance-related challenges to healthcare access highlights the importance of improving geographic accessibility to health services. Regional disparities further emphasize the need for targeted interventions, such as community-based outreach programs to educate women on the benefits of health insurance.

Main text: Introduction : Line 74-79- This paragraph does not add value to your study. The second paragraph almost conveys the same message. I would expect to read some challenges in health financing globally, leading to health insurance as one of the measures to help people access health care services.  It is also essential to establish the global, regional and national context, and then lead the literature review to the local context. The authors have included the later. There is good synthesis of the local context. However, it lacks a broader context.

Response: In recent years, global health policy has increasingly focused on achieving universal health coverage (UHC) as a means of ensuring equitable access to healthcare services and protecting individuals from catastrophic out-of-pocket (OOP) healthcare expenses [1-3]. Health financing remains a significant challenge worldwide, particularly in low- and middle-income countries (LMICs), where high OOP expenditures often hinder access to essential healthcare services and push millions of households into poverty each year [4]. To address these challenges, health insurance has emerged as a critical mechanism to improve access to healthcare, reduce financial barriers, and promote equity in health systems. However, despite its potential, health insurance coverage remains uneven globally, with significant gaps in enrollment, particularly among vulnerable populations such as women, children, and those in low-income settings [5].

In sub-Saharan Africa (SSA), health insurance policies have been implemented over the past two decades as part of broader efforts to achieve UHC and meet the Sustainable Development Goals (SDGs), particularly SDG 3.8 (achieving UHC) and SDG 3.1 (reducing maternal mortality) [6]. Countries such as Ghana, Rwanda, Kenya, Nigeria, and South Africa have introduced various health insurance programmes to improve healthcare accessibility and reduce financial strain on their populations [7]. These programmes have targeted both formal sector employees and informal sector workers, with some extending coverage to entire communities. Despite these efforts, enrollment rates in many SSA countries remain low, with significant disparities in coverage between urban and rural populations, and between men and women [8]. Women, particularly those in their reproductive years, face unique barriers to enrollment, including economic constraints, limited awareness, and sociocultural factors that restrict their access to healthcare services [9].

Sierra Leone, a low-income country in West Africa with a population of approximately 7.9 million, exemplifies these challenges. Women, who constitute 51% of the population, bear a disproportionate burden of healthcare needs, particularly related to maternal and reproductive health [10]. The country continues to face a high maternal mortality ratio, recorded at 717 deaths per 100,000 live births in 2019, and a high adolescent birth rate of 102 births per 1,000 women aged 15–19 [11]. Despite the introduction of health insurance policies in Sierra Leone, enrollment remains persistently low, particularly among women, posing a significant barrier to achieving UHC and improving maternal health outcomes. Understanding the factors that contribute to low health insurance enrollment among women is critical for addressing these disparities and advancing health equity in Sierra Leone.

Line 101-102- I think health insurance goes beyond just reducing maternal mortality, unless it is restricted in the country. Please check if the guideline says that only maternal health services are made available or it also extends to universal health care/primary health care.

Response: Health insurance is a vital strategy for ensuring universal access to affordable and quality healthcare, particularly for vulnerable groups like women and children [12]. While maternal health services are often prioritized in low-income countries such as Sierra Leone due to high maternal mortality rates, health insurance schemes typically aim to provide broader coverage, including universal and primary health care services [12]. These services encompass preventative care, treatment for various diseases, and essential health interventions. In Sierra Leone, health insurance supports the broader goal of universal health coverage, making it crucial to address barriers to enrollment among women to improve overall health outcomes and advance equity in healthcare access [12].
Line 124-125: 'However, health insurance coverage and impact in Sierra Leone is very low, especially among women'. Please add data on overall coverage, and brief comparison of men and women to support your claim.

Response: Previous research on health insurance in Sierra Leone has focused on various aspects, such as the willingness to pay for health insurance in the informal sector [15] and population characteristics influencing the benefits basket of the national social health insurance scheme [16]. Health insurance has been shown to improve health outcomes and reduce poverty among women and their families in Sierra Leone [12]. It reduces out-of-pocket payments for healthcare, increases the utilisation of preventive and curative services, and enhances the quality and efficiency of healthcare delivery [17]. However, health insurance coverage in Sierra Leone remains alarmingly low. According to the 2019 Sierra Leone Demographic and Health Survey (SLDHS), only 4% of women and 3% of men are enrolled in health insurance schemes, highlighting a critical disparity in access to financial protection for healthcare [11]. This low coverage reflects significant barriers to enrollment, particularly for women, who face unique social, economic, and cultural challenges.

Line 125-126- The authors also need to establish the literature gap. Why is there a need to conduct this study? I am sure authors have gone through numerous articles and identified the gap. Please summarize briefly.

Response: Despite the evidence on the potential benefits of health insurance, there is limited research examining the factors contributing to the low enrollment of women in health insurance schemes in Sierra Leone. Existing studies have largely focused on willingness to pay or general population characteristics without delving into the gender-specific barriers that women face [15, 16]. Furthermore, while health insurance is widely recognized as a pro-poor measure, there is a lack of comprehensive analysis on its gendered impact and the specific challenges women encounter in accessing health insurance in low-income settings like Sierra Leone. This study addresses this gap by exploring the factors influencing the low enrollment of women in health insurance using the 2019 SLDHS. The findings will contribute to the existing literature by providing gender-specific insights and evidence-based recommendations to improve health insurance coverage and, ultimately, the health and well-being of women and their families in Sierra Leone.
Methods
Line 133-145: Please mention which name of the data set. DHS study is quite unique that it has multiple datasets and each dataset has its own strength. It is essential to ensure transparency. I also believe the authors have used 'the Guide to DHS statistics' which clearly outlines the dataset that needs to be analysed for certain variables.

Response: The 2019 SLDHS used for this study is part of a series of periodic cross-sectional surveys designed to collect data on demographic, health, and nutritional factors among non-elderly adults and children. The most recent 2019 SLDHS was conducted over four months, from May 2019 to August 2019 [18]. The survey employed a stratified, two-stage cluster sampling design. In the first stage, 578 enumeration areas (EAs) were selected, comprising 214 urban and 364 rural regions. In the second stage, 24 households were systematically selected from each EA, resulting in a total sample size of 13,872 households [18]. For this study, the Individual Recode (IR) dataset from the 2019 SLDHS was used. This dataset is specifically designed to provide detailed information on women aged 15 to 49, including their sociodemographic characteristics, health indicators, and access to healthcare services. The IR dataset was selected because it is the most appropriate for analyzing factors influencing women’s health insurance enrollment. The choice of variables and analysis follows the recommendations outlined in the "Guide to DHS Statistics," which provides a comprehensive framework for identifying the relevant datasets and variables for specific research objectives. The survey collected sociodemographic information from respondents using interviewer-administered questionnaires. A total of 15,574 women aged 15 to 49 were interviewed, and all participants provided written informed consent. Detailed information regarding the sampling technique and survey methodology can be found in the final DHS report [18].

Variables: I believe this can be restructured a bit better. Please use sub-heading- 'Outcome variable' and 'Independent/exploratory variables' so it is easier. It is also essential to provide references to the relevant studies to support how the authors categorised the variables.

Response: **Outcome variable**

The dependent variable in this study was health insurance coverage. This was derived from the question, 'Does any health insurance cover you?' It was coded 1 = “Yes” and 0 = “No”. However, to derive health insurance non-enrollment, we recoded the response options as "1=no" and "0=yes" in the final analysis, where "no" referred to women who were covered by health insurance and "yes" to those who were not enrolled on health insurance.

**Explanatory variables**

We included sixteen explanatory variables in the study. These variables consisted of age classified in a 5–year grouping and categorised as 15–19 = 1, 20–24 = 2, 25-29=3, 30-34=4, 35-39=5, 40-44=6, 45-49=7. Levels of education were captioned as no education = 1, primary = 2, secondary = 3, and higher education = 4. Employment status was categorised as unemployed= 0 and employed =1. Wealth status was categorized as poorest = 1, poorer = 2, middle = 3, richer = 4, and richest = 5. The sex of household head was categorised as male=1 and female=2. Marital status was categorized as never in union =1, married =2, cohabitating =3, widowed =4, divorced =5, and separated =6. The total number of children born was categorised as zero birth=0, one to three births=2 and Four or more births=3. Challenges with getting medical help for self (i.e., getting permission to go, getting the money needed for treatment, and distance to health facility) were categorised as a big problem =1 and not a big problem=2. The frequency of reading newspapers, listening to radio, and viewing television was not at all = 1, less than once a week = 2, and at least once a week = 3. Place of residence was categorised as Urban=1 and Rural=2. Region captured as Eastern=1, Northwestern=2 Northern=3, Southern=4, and Western=5. The selection of the explanatory variables was guided by their presence in the DHS datasets and their association with health insurance coverage, as indicated by prior research [5, 21–23].

Statistical analysis- as mentioned earlier- it is essential to use standard statistical method which authors have done. Please use the same term consistently.
Line 185-189: This can be shortened . 'Weighted analysis was conducted using XX command in Stata. 
Response: Finally, we fitted a multiple logistic regression analysis to examine the factors associated with health insurance non-enrollment coverage by including all socioeconomic and demographic predictors (Table 2). Weighted analysis was conducted using the "svyset" command in Stata version 14 to account for disproportionate sampling, non-response, and the complex survey design of the DHS data. Statistical significance was set at p-values less than 0.05.
Results: Table 1. looks very busy. It is needless to report both weighted and unweighted frequency distribution. I suggest to report weighted column only.  In the same table though, the health insurance part does not have frequency distribution. Since the enrolment rate is very low, it is essential to see the frequency distribution too. I believe the authors proceed with binary logistic regression without performing chi-square test. If that is the case, the reason has to be explained in  analysis section.

Response: We appreciate the reviewer’s comments and have made the necessary revisions to Table 1. We have removed the unweighted frequency distribution and retained only the weighted frequency distribution, as the weighted data better reflect the population estimates and align with the recommendations of the DHS guidelines.

Regarding the health insurance frequency distribution, we acknowledge the importance of understanding the enrollment rates. However, given that the primary focus of this study is to explore the factors influencing non-enrollment in health insurance, we opted to present the percentage distribution of health insurance coverage (Yes/No) across various sociodemographic and socioeconomic characteristics, as this provides a clearer picture of the patterns associated with non-enrollment. Including the frequency distribution for health insurance enrollment would not add substantial value to the analysis, given the already low enrollment rates, and could make the table unnecessarily lengthy and harder to interpret.

As for the chi-square tests, we did not perform them because our primary objective was to identify the predictors of health insurance non-enrollment using binary logistic regression. Logistic regression is a robust analytical method that allows us to control for potential confounders and assess the independent association of each variable with the outcome of interest. While chi-square tests are useful for bivariate analyses, they do not account for confounding variables, which are critical in understanding the multifactorial nature of health insurance non-enrollment. Therefore, we proceeded directly with logistic regression to ensure a more comprehensive and adjusted analysis.
Line 207- 'Predictor' is a very strong word. With a cross sectional study such as DHS, we can not ascertain predictors. The most we can do is to explore factors associated. Please revise the heading accordingly. Similar to what I mentioned earlier, the authors need to present result in one direction. 'Non-enrolment' is already a negative outcome. Double negative in results has made it difficult to understand.

Response: The results in Table 2 indicate that several factors are significantly associated with non-enrollment in health insurance among women in Sierra Leone after adjustment for other variables. Women with higher education were significantly less likely to be uninsured (aOR: 0.35, 95% CI: 0.18-0.64, p=0.001) compared to those with no education, indicating that higher education was protective against non-enrollment. Employment also reduced the odds of being uninsured (aOR: 0.47, 95% CI: 0.36-0.62, p<0.001) compared to unemployment. Listening to the radio less than once a week was associated with lower odds of being uninsured (aOR: 0.71, 95% CI: 0.53-0.97, p=0.032) compared to not listening at all. On the other hand, women who reported distance to a health facility as a big problem were more likely to be uninsured (aOR: 2.21, 95% CI: 1.03-4.75, p=0.041) compared to those who did not consider it a problem. Regionally, women living in the Northwestern (aOR: 0.07, 95% CI: 0.03-0.14, p<0.001) and Northern regions (aOR: 0.28, 95% CI: 0.12-0.66, p=0.004) were significantly less likely to be uninsured compared to those in the Eastern region.

Table 2- Model 1 and Model 2; This is misleading. Please just write: Crude Odds Ratio ( 95% Confidence Interval), and Adjusted Odds Ratio ( 95% Confidence Interval). Model 1 is not actually one model. It is run separately for each independent variable. P-value  should be: p-value cOR: it is alright to mention: odds ratio and Adjusted odds ratio to make it more readable.

Response: We have made the revision in the manuscript.
Table 1- is very long.  I would suggest- keep the entire table in supplementary file.  And, only the significant results  in the main file. It will improve the readability of the manuscript. Those who are interested in detailed results can always refer to supplementary files.

Response: Thank you for the reviewer’s suggestion regarding Table 1. We have revised the table by removing the unweighted frequencies, which has reduced its length and improved its readability. However, we believe it is important to retain Table 1 in the main manuscript rather than moving it to the supplementary file for several reasons. First, the table provides essential context by presenting the distribution of health insurance coverage across key sociodemographic and socioeconomic characteristics, which is critical for understanding the study findings. Second, keeping the table in the main manuscript ensures transparency and accessibility, allowing readers to easily reference the data without needing to consult supplementary materials. Finally, the revised table is now more concise and directly relevant to the study's objectives, supporting the narrative without being overly lengthy.

Please re run multiple logistic regression. If you have screened the association using binary logistic regression, you need to include only significant variables in subsequent multiple logistic regression. For instance, 'total number of children ever born'- is not significant in binary regression- but has been included in multiple logistic regression.  
Response: We have now re-run the multiple logistic regression analysis in the revised manuscript.
Line 208- onwards needs to change after the analysis. List the variables which were significant in binary logistic regression. Then explain those which were significant in multiple logistic regression.

Response: The results in Table 2 indicate that several factors are significantly associated with non-enrollment in health insurance among women in Sierra Leone after adjustment for other variables(education level, employment status, frequency of reading newspaper or magazine, listening to radio, watching television, permission to go, distance to a health facility,not wanting to go alone, wealth status, residence and region) . Women with higher education were significantly less likely to be uninsured (aOR: 0.35, 95% CI: 0.18-0.64, p=0.001) compared to those with no education, indicating that higher education was protective against non-enrollment. Employment also reduced the odds of being uninsured (aOR: 0.47, 95% CI: 0.36-0.62, p<0.001) compared to unemployment. Listening to the radio less than once a week was associated with lower odds of being uninsured (aOR: 0.71, 95% CI: 0.53-0.97, p=0.032) compared to not listening at all. On the other hand, women who reported distance to a health facility as a big problem were more likely to be uninsured (aOR: 2.21, 95% CI: 1.03-4.75, p=0.041) compared to those who did not consider it a problem. Regionally, women living in the Northwestern (aOR: 0.07, 95% CI: 0.03-0.14, p<0.001) and Northern regions (aOR: 0.28, 95% CI: 0.12-0.66, p=0.004) were significantly less likely to be uninsured compared to those in the Eastern region.

Table 2: Current marital status  - Please re-categorise the variable. There '1' observation in one category, yet it is included as is. There is no reason to use  ( ) and [ ] to indicate the odds ratio. It is acceptable to use (  ) as there is always a column heading in the table.

Response: Thank you for the reviewer’s observation regarding the categorization of the marital status variable.We mistakenly thought divorced was the reference category. We have now added the crude odds ratio and p value so there is no need to recategorize.
Discussion: This is extremely long discussion section. This needs to be shortened significantly and just focus on your aim. This might change after re-analysis. Please edit/rewrite as appropriate after you have the new results but ensure that it is not long.

Response: We have now carefully revised the discussion section in the manuscript.

Reference: Ref #1,12, 14,16 and more- there are significant issues. Please ensure it is according to the journal guidelines.

Response: We have now revised the entire references as per PLOS One guidelines.

Reviewer #2: Dear authors,
Thank you for reviewing the study entitled “ Determinants of Non-enrollment on Health Insurance among Women in Sierra Leone: a cross-sectional analysis of the 2019 Sierra Leone Demographic Health Survey." This topic is important; however, the study has some shortcomings. I have some comments that I hope the authors will take into account in improving the manuscript.

Response: Thank you.

Abstract: I find that your summary is very long.

Response: Thank you. We have revised our summary but its still within the 500 words as per PLOS One abstract guidelines.
Introduction:
Your introduction is well-structured and contains all the necessary information about health insurance. I congratulate you on that. However, some remarks have been raised:
- Support your study with other results related to the effect of financial inaccessibility due to the lack of universal health coverage on the sexual and reproductive health of women of childbearing age ;
Include references for the information presented. Exemple : Line 101: include references for the information presented on Sierra Leone.

Response: We have thoroughly revised the introduction and also included references were necessary.
Materials and Methods:
Your method is excellently designed. Well done.

Response: Thank you.
Results:
- I invite you to present the main results associated with the lack of enrollment in health insurance according to various variables related to the sociodemographic and socioeconomic characteristics of women in Sierra Leone.

Response: Table 1 shows the results of non-enrollment in health insurance among women in Sierra Leone based on various sociodemographic and socioeconomic characteristics. Younger women (aged 15-19) exhibit the highest proportion of non-enrollment (97.09%), with coverage slightly improving with age but remaining low overall. Women with higher education levels are more likely to have insurance (14.30% coverage), while those with no education face the highest non-enrollment (96.44%). Married women show slightly better coverage (4.26%) compared to those never in union (3.57%), while divorced women have no coverage at all. Employment status influences coverage, with employed women having higher enrollment (4.54%) compared to unemployed women (2.75%). Wealth disparities are evident, as the richest women have the highest coverage (6.04%), while the poorest have the lowest (1.88%). Urban residents (5.12%) are more likely to have coverage than rural residents (3.01%). Regional differences are stark, with the Northwestern region having the highest coverage (10.29%) and the Eastern region the lowest (0.91%). Access to information and healthcare barriers also play a role; women who frequently read newspapers or watch television and those who face fewer obstacles in accessing healthcare report higher coverage rates
- Please review the percentages in the tables. If they are above or below 100%. For example, in Table 1: Current marital status, Married.

Response: All categories across variables now sum to 100%
Discussion
You discussed your results well. However, it will be stronger if supported by other studies in similar contexts.

Response: We have now carefully revised the discussion section in the manuscript.

Reviewer #3: METHODOLOGY

Strengths:

Clear Sampling Design: The study uses a well-organized sampling method called stratified two-stage cluster sampling, which is reliable for large surveys. The process of selecting households and enumeration areas is clearly explained, making the sampling process transparent.
Response: Thank you.

Detailed Variable Description: The variables used in the study are well-defined, covering a wide range of factors like sociodemographic and health-related details. This strengthens the analysis, particularly when looking at health insurance non-enrollment.

Response: Thank you.

Thorough Statistical Analysis: The study uses both descriptive and inferential statistics, including logistic regression, which is appropriate for its goals. The report also checks for issues like multicollinearity, ensuring that the model used is accurate.

Response: Thank you.

Following Guidelines: The study follows the STROBE guidelines, which adds to its credibility by ensuring methodological rigor.
Response: Thank you.
Areas for Improvement:

Ethical Considerations: While the study mentions informed consent, more details on ethical aspects like data confidentiality and protection of participants would improve transparency. Information on institutional ethical approval should also be added.

Response: The MEASURE DHS program adheres to rigorous international ethical standards to ensure the protection and confidentiality of survey participants. As part of the 2019 Sierra Leone Demographic and Health Survey (SLDHS), ethical approval for the survey protocol was obtained from the Sierra Leone Ethics and Scientific Review Committee and the ICF Institutional Review Board. These institutions ensure compliance with ethical guidelines, including data confidentiality and participant protection. All participants provided written informed consent prior to participation, and for minors, consent was obtained from their legally authorized representatives. During the survey, data confidentiality was strictly maintained through anonymization, ensuring that no personally identifiable information was included in the publicly available dataset. For our study, the dataset was accessed from the MEASURE DHS website after obtaining appropriate permissions, and no additional ethical clearance was required as this was a secondary analysis of de-identified, publicly available data. Further details on the ethical procedures employed by the DHS program, including measures to protect the privacy of respondents, can be found at [https://dhsprogram.com/Methodology/Protecting-the-Privacy-of-DHS-Survey-Respondents.cfm](https://dhsprogram.com/Methodology/Protecting-the-Privacy-of-DHS-Survey-Respondents.cfm).

Clarifying Explanatory Variables: Some variable categories, like wealth or marital status, need more explanation. For example, defining what makes someone "poorest" or "richest" would add clarity.

Response: We included sixteen explanatory variables in the study, selected based on their availability in the DHS datasets and their established association with health insurance coverage in prior research [5, 21–23]. The variables and their categorization are as follows:

1. Age: Classified in 5-year groups and coded as 15–19 = 1, 20–24 = 2, 25–29 = 3, 30–34 = 4, 35–39 = 5, 40–44 = 6, and 45–49 = 7.

2. Education level: Categorized as no education = 1, primary = 2, secondary = 3, and higher education = 4, based on the highest level of formal education attained by the respondent.

3. Employment status: Categorized as unemployed = 0 and employed = 1, based on the respondent’s self-reported engagement in any form of income-generating activity during the survey period.

4. Wealth index: Categorized as poorest = 1, poorer = 2, middle = 3, richer = 4, and richest = 5. The wealth index is a composite measure of a household’s cumulative living standard, calculated by the DHS using principal component analysis. It is based on household ownership of selected assets, materials used for housing construction, and access to water and sanitation facilities. Households are then ranked and divided into five quintiles, with the "poorest" representing the lowest quintile and "richest" representing the highest quintile.

5. Sex of household head: Categorized as male = 1 and female = 2, based on whether the head of the household is male or female.

6. Marital status: Categorized as never in union = 1, married = 2, cohabiting = 3, widowed = 4, divorced = 5, and separated = 6. These categories reflect the respondent’s self-reported marital situation at the time of the survey.

7.Total number of children ever born: Categorized as zero births = 0, one to three births = 2, and four or more births = 3, based on the total number of live births reported by the respondent.

8. Challenges with getting medical help for self: Three dimensions were assessed getting permission to go, getting the money needed for treatment, and distance to the health facility. Each was categorized as a big problem = 1 and not a big problem = 2, based on the respondent’s perception of these barriers.

9.Frequency of media exposure: The frequency of reading newspapers or magazines, listening to the radio, and watching television was categorized as not at all = 1, less than once a week = 2, and at least once a week = 3, based on the respondent’s self-reported media consumption habits.

10. Place of residence: Categorized as urban = 1 and rural = 2, based on the DHS classification of the respondent’s living area.

11.Region: Categorized as Eastern = 1, Northwestern = 2, Northern = 3, Southern = 4, and Western = 5, corresponding to the geographic regions of Sierra Leone where the respondents reside.

Handling Missing Data: The study uses methods to adjust for sampling and non-response but could provide more information on how it handled missing data, a common issue in large surveys.

Response: There were no missing data as the analysis included all women 15,574 who took part in the 2019 SLDHS.

Dependent Variable Coding: The recoding of health insurance enrollment (1 = no, 0 = yes) could be confusing. Explaining why this approach was used would clarify things for readers.

Response: The dependent variable in this study was health insurance coverage, derived from the question, "Does any health insurance cover you?" with the original response options coded as 1 = “Yes” and 0 = “No.” For the purpose of our analysis, we recoded the variable to reflect health insurance non-enrollment, where 1 = “No” (not covered by health insurance) and 0 = “Yes” (covered by health insurance). This approach was used to align the coding with the primary focus of the study, which was to identify factors associated with the lack of health insurance coverage. By coding "no enrollment" as 1, we were able to make the interpretation of the results more intuitive, as higher values for the dependent variable corresponded to the outcome of interest (non-enrollment). This recoding ensures clarity in the analysis and facilitates a consistent interpretation of the results in relation to predictors of health insurance non-enrollment.

Presentation of Results: While the study uses odds ratios (aOR) appropriately, it would help to mention how they checked that the model was a good fit, using tests like goodness-of-fit.

Response: To ensure the robustness of our results, we assessed its goodness-of-fit using the Hosmer-Lemeshow test, which confirmed that the model was a good fit for the data (p > 0.05).

Justifying Variable Selection: The study selects explanatory variables based on past research, but it could elaborate on why specific variables (e.g., region, employment) were chosen and how these have been linked to health insurance in previous studies.

Response: The variables included were carefully chosen based on their availability in the DHS datasets and their demonstrated relevance in prior research examining factors associated with health insurance coverage. For example, region was included because geographic disparities often play a significant role in access to health services and insurance coverage, with prior studies highlighting regional differences in infrastructure, healthcare accessibility, and socioeconomic conditions that influence enrollment. Similarly, employment status was selected because it is a key determinant of financial stability and access to employer-sponsored health insurance programs, as supported by evidence from studies showing higher insurance enrollment among employed individuals compared to those unemployed. Other variables, such as education level, wealth index, and marital status, were included due to their well-established associations with health-seeking behavior and financial capacity to enroll in insurance schemes. By including these variables, we aimed to comprehensively capture the multidimensional factors influencing health insurance coverage.

Analysis Software: Stata 14 is a good software choice, but it might be useful to explain why this version was used, as newer versions offer improved features.

Response: All revised analysis have now been conducted in Stata version 18.

Overall: The methodology section is detailed and could be replicated. However, providing more information on ethics, defining variables more clearly, and explaining some choices (like recoding) would make it even stronger.

Response: We have now revised and addressed all your comments.

RESULTS

Clarity and Structure: The results section is repetitive in some parts, especially when discussing percentages of health insurance non-enrollment. Simplifying the language and making it more concise would improve readability. Also, ensure that all figures and tables are labeled correctly and placed in the right sections of the text.

Response: We have now carefully revised the results section in the manuscript.


Descriptions of Tables: The transition from figures to tables can be smoother. For example, before introducing Table 1, provide a brief explanation of what the table will show, such as a breakdown of sociodemographic factors.

Response: We have now carefully revised the results section in the manuscript.

Interpreting the Data: When discussing variations in non-enrollment, be specific about which variables show significant differences (e.g., age, education). Highlight key findings so readers don’t have to sift through all the data themselves.

Response: We have now carefully revised the results section in the manuscript.

Predictors Section: The analysis of predictors is comprehensive, but the significant findings should be emphasized more clearly. For example, when discussing differences between regions, mention the exact odds ratios to give readers a clearer picture.

Response: We have now carefully revised the results section in the manuscript.

Terminology and Precision: Avoid vague language. For example, instead of saying, “There are variations in the distribution of explanatory variables,” specify which variables vary, like age or education.

Response: We have now carefully revised the results section in the manuscript.

Contextualizing Findings: Provide some insight into why certain groups are more likely to be uninsured. Although this is mainly for the discussion, a brief mention here would prepare readers for the interpretation later.

Response: We have now carefully revised the results section in the manuscript.

DISCUSSION

Strengths:

Identification of Key Issues: The discussion identifies important factors that influence health insurance enrollment, such as lack of awareness, financial barriers, and differences between regions.

Response: Thank you.

Context with Other Studies: Similar findings from other studies are used to contextualize the results within the broader literature, which is a good approach.

Response: Thank you.

Policy Implications: The discussion suggests practical policy solutions, such as outreach programs and financial incentives, to increase health insurance coverage.

Response: Thank you.

Areas for Improvement:

Repetitiveness: Some points, like financial barriers and lack of awareness, are repeated. These can be consolidated to improve the flow of the discussion.

Response: We have now carefully revised the discussion section in the manuscript.


Depth of Analysis: Some explanations need more evidence. For example, linking low media exposure to income levels feels speculative. Explore cultural and social factors in more depth, like how local beliefs affect health insurance enrollment.

Response: We have now carefully revised the discussion section in the manuscript.


Inconsistencies in Findings: Some findings seem contradictory, such as why women with higher education enroll less often. More explanation is needed to clarify these points.

Response: We have now carefully revised the discussion section in the manuscript.


Discussion Structure: Group related factors under clear subheadings (e.g., "Socioeconomic Barriers") to make the discussion easier to follow.

Response: We have now carefully revised the discussion section in the manuscript.


Data Limitations: The limitations section could elaborate more on the study’s cross-sectional design and how future research could address this.

Response: This study addresses a critical issue access to health insurance as a pathway to achieving universal health coverage and improving women’s well-being. While the study benefits from the use of a large, nationally representative SLDHS and standardized data collection procedures, several limitations should be acknowledged.

First, the cross-sectional design of the study limits our ability to establish causal relationships between the explanatory variables and health insurance non-enrollment. While associations were identified, it is not possible to determine the directionality or temporality of these relationships. For instance, while higher education and employment were associated with lower odds of being uninsured, it is unclear whether these factors directly lead to increased enrollment or whether other unmeasured variables play a role. Future research could address this limitation by employing longitudinal study designs to better understand how changes in socioeconomic and demographic factors influence health insurance enrollment over time.

Second, the SLDHS dataset, while robust, does not provide information on the type of health insurance schemes (e.g., public or private), limiting our ability to analyze whether specific types of insurance are more accessible or effective for women. Future studies could explore this distinction to provide more targeted recommendations for improving health insurance coverage.

Finally, while the study highlights key socioeconomic and geographic factors influencing health insurance non-enrollment, it does not fully account for the cultural and social factors that may shape women's decisions to enroll in health insurance. These factors may include perceptions of health insurance, trust in healthcare systems, and traditional norms, which are difficult to capture in a cross-sectional survey. Qualitative or mixed-methods research could provide deeper insights into these contextual factors and help design culturally appropriate interventions to promote health insurance enrollment.

Policy Recommendations: The policy suggestions could be more detailed. For example, explain how financial incentives or mobile technology could help increase enrollment in rural areas.

Response: The findings of this study highlight critical areas for policy and programmatic interventions to improve health insurance enrollment among women. First, the protective effect of higher education against health insurance non-enrollment underscores the need to invest in women’s education as a long-term strategy to enhance health insurance coverage. Policies aimed at improving access to education, particularly for girls and women, could have downstream benefits for health insurance uptake and overall health outcomes. Second, the significant association between employment and reduced odds of being uninsured suggests that economic empowerment programs and initiatives to formalize employment, particularly for women, could play a pivotal role in increasing health insurance enrollment. Policymakers should consider expanding employer-sponsored insurance schemes and creating incentives for informal sector workers to join national health insurance programs.

Additionally, targeted health communication strategies, such as leveraging radio as a medium for raising awareness about health insurance, could be effective, especially for women who are already engaging with this medium. Programs that promote regular exposure to health-related messages through radio broadcasts could help bridge the knowledge gap and encourage enrollment. The finding that distance to health facilities increases the likelihood of non-enrollment highlights the need for policies that address geographic barriers to healthcare access. Investments in infrastructure and the establishment of health facilities in underserved areas could reduce these barriers and encourage insurance enrollment by increasing the perceived utility of health coverage.

Finally, the regional disparities observed in health insurance enrollment emphasize the importance of region-specific interventions. Policymakers should prioritize tailored interventions in regions with low enrollment rates, such as subsidies, awareness campaigns, and outreach programs, to address the unique challenges faced by women in these areas.

Conclusion: The conclusion could summarize key findings more directly and offer a future research agenda to address the study's limitations.

Response: This study reveals a high proportion of health insurance non-enrollment among women (96.0%), highlighting a critical gap in achieving universal health coverage. Key findings indicate that higher education and employment significantly reduce the likelihood of being uninsured, underscoring the importance of socioeconomic empowerment in driving health insurance enrollment. Additionally, listening to the radio was associated with lower odds of non-enrollment, suggesting the potential of targeted health communication strategies to promote awareness and uptake. Conversely, geographic barriers, such as distance to health facilities, were associated with higher odds of being uninsured, emphasizing the need for improved healthcare infrastructure and accessibility. Regional disparities were also evident, with women in the Northwestern and Northern regions significantly more likely to be insured compared to those in the Eastern region, calling for region-specific interventions to address inequities.

These findings suggest that policymakers should prioritize education, employment opportunities, accessible healthcare infrastructure, and targeted health communication to improve health insurance enrollment among women. Future research should explore additional barriers to health insurance enrollment, such as cultural factors, affordability, and the quality of insurance schemes, to provide a more comprehensive understanding of the challenges. Furthermore, longitudinal studies could help assess the long-term impact of interventions aimed at increasing health insurance coverage and reducing disparities across different regions and population groups. Addressing these gaps will be critical for achieving equitable and sustainable health insurance coverage for all women.

We hope that we have adequately addressed the reviewers' comments, and we look forward to receiving a favorable outcome on our paper.

Yours Sincerely,

Augustus Osborne

Corresponding Author
