# Supplementary material for: Health insurance non-enrollment among women in Sierra Leone: A cross-sectional analysis of the 2019 Demographic and Health Survey
Source: PLoS One. 2025 Jul 31;20(7):e0323538. doi: 10.1371/journal.pone.0323538 (PMC12312921; doi:10.1371/journal.pone.0323538)
Supplement: Attachment — Submitted filename: Response_to_Reviewers_auresp_2.docx [file pone.0323538.s003.docx]

The Editor

PLOS ONE

1^st^ April 2025

Ref: PONE-D-24-25104

**Title:** Determinants of Non-enrollment on Health Insurance among Women in Sierra Leone: a cross-sectional analysis of the 2019 Sierra Leone Demographic Health Survey.

Response to Reviewers' comments

Dear Sir/Madam,

We want to express our sincere thanks for painstakingly reviewing our manuscript and providing valuable comments and suggestions. Please see our point-by-point response to the reviewers' comments and suggestions. Revisions are highlighted with track changes in the revised manuscript.

**ACADEMIC EDITOR:**

There are a few minor comments from the reviewers. I now invite the authors to respond to the comments and make corrections as necessary. I suggest that the authors change 'determinants' in their title to 'factors associated with ...,' as 'determinants' can imply causation, which is not the case in the current study. Additionally, the authors should thoroughly proofread their work (line by line) and fact-check every statement to ensure smooth progress without delay. I look forward to receiving the revised manuscript.

Response: Thank you. We have now changed the title to factors associated with and have have thoroughly proofread the article line by line to ensure smooth progress without delay.

Reviewer #1: The authors have responded the majority of my suggestion. I did not review the last version discussion as the results were likely to change. I have provided some minor suggestions for authors to address. 
Response: Thank you.
Abstract: The authors have used aOR with p- value. Since 95% CI is reported, it is needless to report p-values
Response: Thank you. We have now deleted the p values in the abstract.
Introduction, Line 198:  only 4% of women and 3% of men are enrolled in health insurance schemes ... was added in response to earlier feedback ' 'However, health insurance coverage and impact in Sierra Leone is very low, especially among women'. Please add data on overall coverage, and brief comparison of men and women to support your claim.'  If we look at the revised version, women have higher enrolment than men. How do you justify the research gap is still an issue. 
Response: Health insurance coverage in Sierra Leone remains alarmingly low, with only 4% of women and 3% of men enrolled in health insurance schemes, according to the 2019 Sierra Leone Demographic and Health Survey (SLDHS) [11]. Overall, this translates to just 3.5% of the population having access to health insurance, underscoring the critical lack of financial protection for healthcare [11]. While women have a slightly higher enrollment rate than men, this marginal difference does not negate the significant barriers to enrollment faced by both genders, particularly women. Women in Sierra Leone encounter unique social, economic, and cultural challenges, such as lower income levels, reduced decision-making power, and limited access to information about health insurance schemes [12]. These factors exacerbate disparities in healthcare access and financial security, making the low overall coverage and the gendered barriers to enrollment a pressing issue for policymakers and researchers alike.
Explanatory variables: Great to see a better explanation of the explanatory variables. I advise authors to  structure this in paragraphs, not in numbering that is usually done in powerpoints. 
Response: We included sixteen explanatory variables in the study, selected based on their availability in the DHS datasets and their established association with health insurance coverage in prior research [5, 21–23]. These variables were categorized into distinct groups to facilitate analysis.

Age was classified into seven 5-year groups: 15–19, 20–24, 25–29, 30–34, 35–39, 40–44, and 45–49. Each group was coded numerically from 1 to 7, respectively. Education level was categorized based on the highest level of formal education attained by the respondent: no education (1), primary (2), secondary (3), and higher education (4). Employment status was determined based on respondents’ self-reported engagement in income-generating activities during the survey period, categorized as unemployed (0) and employed (1).

The wealth index, a composite measure of a household’s cumulative living standard calculated by the DHS using principal component analysis, was divided into quintiles: poorest (1), poorer (2), middle (3), richer (4), and richest (5). This index considers household ownership of selected assets, housing construction materials, and access to water and sanitation facilities. Sex of household head was categorized as male (1) or female (2), while marital status was classified into six categories: never in union (1), married (2), cohabiting (3), widowed (4), divorced (5), and separated (6), based on respondents’ self-reported marital situation.

The total number of children ever born was categorized into three groups: zero births (0), one to three births (2), and four or more births (3). We also assessed challenges with getting medical help for oneself across three dimensions: getting permission to go, obtaining the money needed for treatment, and distance to the health facility. Each dimension was categorized as a big problem (1) or not a big problem (2), based on respondents’ perceptions.

Frequency of media exposure was measured by how often respondents read newspapers or magazines, listened to the radio, or watched television. This was categorized as not at all (1), less than once a week (2), and at least once a week (3). The place of residence was classified as urban (1) or rural (2), based on the DHS classification of the respondent’s living area. Lastly, region was categorized into five geographic areas of Sierra Leone: Eastern (1), Northwestern (2), Northern (3), Southern (4), and Western (5).
Results sections referring to Table 1;  "Table 1- is very long." in response to these authors have suggested the unweighted frequencies are removed which is good. The table is 4 page long in its current form which is very long for any publication. I would have kept the long table in a supplementary file, and the rest in main file. It still ensures transparency as it comes with the main article. At times the Authors have to be kind to readers as well. Making articles very long is extremely unhelpful.  I will leave it to the academic editor to make a decision.
Response: Thank you for your feedback regarding the length of Table 1. We understand that the current formatting makes the table appear long in the manuscript. However, the length is primarily due to the double-spacing and layout requirements for submission. Once the manuscript is formatted for publication by PLOS ONE, the table will fit within a single page or at most two pages, making it much more concise and reader-friendly.

We believe that keeping Table 1 within the main manuscript is important for transparency and accessibility, as it provides critical details on the distribution of health insurance coverage across various sociodemographic and socioeconomic characteristics. Including this table in the main file ensures that readers have immediate access to this information without needing to refer to supplementary files, which can sometimes disrupt the flow of reading.

Results section referring to Table 2: The effect sizes are written (aOR: 0.35, 95% CI: 0.18-0.64, p=0.001). Please remove p-value throughout as that authors have written 95% CI. which is the better measure to show the association. 
Response: Thank you. We have now deleted the p values in the results.
Discussion: Line 344-353: Those who listen to radio less than a week had lower odds compared but not those who did so once a week. I think this result is very hard to justify. You argument that listening to the radio as protective for health insurance enrolment is plausible but your result does not support. May be try merging the categories 'not at all' vs 'listens to radio (less than a week or at least once a week). If you get a result where listening to the radio comes with less likelihood of non-enrollment, your current discussion can support. Alternatively, the discussion has to reflect what you have in table 2. 
Response: Our study found that women who listen to the radio less than once a week have significantly lower odds of non-enrollment in health insurance compared to those who do not listen to the radio at all, while women who listen at least once a week also showed lower odds, though this association was not statistically significant in the adjusted model. These findings suggest that listening to the radio, regardless of frequency, may increase awareness and understanding of health insurance, potentially facilitating enrollment. This aligns with findings from Amu et al. [5] in SSA, which highlight the role of media exposure in promoting health-related behaviors. Women who listen to the radio may be exposed to health messages or campaigns emphasizing the benefits of health insurance, even with limited frequency. However, the association may also reflect underlying socioeconomic factors, as women who listen to the radio may have higher education or income levels, enabling them to better understand and afford health insurance [28]. Conversely, women who do not listen to the radio may rely on alternative sources of information, such as community health workers or family networks, to support their decision-making [30,33]. These findings underscore the importance of media exposure in shaping health insurance enrollment behaviors and suggest that targeted health communication through radio could play a critical role in increasing coverage [34,35].
Line 354-362: Getting medical help for self: distance to a health facility: is important and same old issue. Multiple 'Tyranny of distance' articles have been written. It is not so much about 'quality' and 'trust' it is more about 'access issue' due to various logistical issues. 
Response: In the current study, women who reported having a big problem with the distance to health facilities had higher odds of non-enrollment in health insurance coverage compared to those who did not face such a problem. This finding highlights the critical role of access barriers in shaping health insurance enrollment in Sierra Leone. Women living far from health facilities may face logistical challenges, such as long travel times, limited transportation options, and higher transportation costs, which can hinder their ability to access health services and information about health insurance enrollment [29,33,35]. Additionally, the physical distance may deter women from seeking healthcare altogether, reducing the perceived value of health insurance as they may not see it as a practical or necessary investment if accessing healthcare remains difficult [17]. These logistical and access-related challenges underscore the importance of addressing infrastructural and transportation barriers to improve health insurance coverage, particularly for women in remote areas.
Line: 363: Regional difference is very important for any country. There may be inherent issues due to systemic marginalization of certain region. I would have thought authors provide some contextual information . There is opportunity to make this paragraph really important. Think about transport access, economic and employability opportunities, education, female literacy, gender issues, ethnic variations in the regions that are prone to non-enrolment. There is very important for authors to explore. 
Response: Our results indicate that women living in the Northwestern and Northern regions had lower odds of non-enrollment in health insurance coverage compared to women in the Eastern region of Sierra Leone. This regional difference likely reflects broader systemic disparities and contextual factors influencing health insurance enrollment [30]. The Eastern region has historically faced challenges such as limited transport infrastructure, which restricts access to health facilities and health insurance enrollment points, and fewer economic and employment opportunities, which may reduce the ability of women to afford health insurance [37]. Lower levels of female literacy and education in the Eastern region may also hinder women's understanding of the benefits and processes of health insurance enrollment. Additionally, gender-related issues, such as reduced autonomy in decision-making, could further limit women's ability to prioritize health insurance [30,37]. Ethnic and cultural variations may also play a role, influencing trust in formal health systems and preferences for traditional or informal providers. In contrast, women in the Northwestern and Northern regions may benefit from better transport access, higher literacy rates, and greater exposure to health-related information, enabling them to perceive the value of health insurance and access enrollment services more readily. These findings highlight the need to address systemic marginalization and regional inequities by improving infrastructure, education, and economic opportunities in underserved areas like the Eastern region to enhance health insurance coverage.
Conclusion: Public health implications have been explained in in earlier section. I would suggest authors make the conclusion really short. For example in one paragraph: highlight key points: Very low uptake, and which groups should be the focus of interventions and end with one future research.

Response: This study highlights a very low uptake of health insurance among women in Sierra Leone (96.0% non-enrollment), underscoring a significant barrier to achieving universal health coverage. Women with higher education, employment, and access to health communication via radio were less likely to be uninsured, while those facing geographic barriers, such as distance to health facilities, and those in the Eastern region were more likely to be uninsured. These findings suggest that interventions should prioritize improving education, employment opportunities, healthcare accessibility, and targeted health communication, particularly for women in underserved regions like the East. Future research should focus on exploring cultural and financial barriers to health insurance enrollment to inform more inclusive and effective policies.

Reviewer #2: Dear authors,
Thank you for studying the article entitled “Determinants of Non-enrollment in Health Insurance among Women in Sierra Leone: a cross-sectional analysis of the 2019 Sierra Leone Demographic Health Survey.” This topic is significant, but the study has some shortcomings. I would like to share a few comments that could help improve the manuscript.

Response:
Abstract: I find that the conclusion of your abstract is very long.

Response: Health insurance non-enrollment remains critically high among women in Sierra Leone. Education, employment, and access to health information via radio were associated with increased enrollment, highlighting the need to address socioeconomic barriers and leverage mass media campaigns. Geographic and regional disparities underscore the importance of improving healthcare accessibility and implementing targeted, community-based interventions to promote health insurance uptake.
Introduction:
Your introduction is well-structured and contains all the necessary information about health insurance. I congratulate you on that. However, some remarks have been made:
• Include references for the information presented. For example: line 113: include references for the information presented on the education level of women in Sierra Leone.

Response: Women, particularly those in their reproductive years, face unique barriers to enrollment, including economic constraints, limited awareness, and sociocultural factors that restrict their access to healthcare services [9].

Sierra Leone, a low-income country in West Africa with a population of approximately 7.9 million, exemplifies these challenges. Women, who constitute 51% of the population, bear a disproportionate burden of healthcare needs, particularly related to maternal and reproductive health [10]. The country continues to face a high maternal mortality ratio, recorded at 717 deaths per 100,000 live births in 2019, and a high adolescent birth rate of 102 births per 1,000 women aged 15–19 [11].
Materials and Methods:
Your method is very well written. Well done.

Response: Thank you.
Results:
Your results have been well presented, emphasizing the main findings related to non-enrollment in health insurance according to the various sociodemographic and socioeconomic characteristics of women in Sierra Leone. Great job.

Response: Thank you.
Discussion:
Your discussion identifies the important factors that influence enrollment in health insurance, particularly the lack of awareness, financial barriers, and differences between regions. Well done.

Response:Thank you.
Recommendations:
Avoid repetitions and try to be concise, especially regarding awareness, policy implications, and strategic recommendations.

Response: The findings of this study underscore key policy and programmatic priorities to improve health insurance enrollment among women in Sierra Leone. Investing in women’s education is crucial, as higher education significantly reduces non-enrollment, with long-term benefits for health coverage and outcomes. Similarly, economic empowerment through formalizing employment and expanding employer-sponsored insurance schemes, alongside incentives for informal sector workers, could enhance enrollment. Targeted health communication, particularly via radio, offers a cost-effective strategy to raise awareness and bridge knowledge gaps. Addressing geographic barriers through investments in healthcare infrastructure and expanding services in underserved areas is essential to improve access and the perceived value of insurance. Lastly, region-specific interventions, such as subsidies and localized outreach programs, are necessary to tackle disparities and promote equitable health insurance coverage.
Conclusion: Well written. Great job.

Response: Thank you.

We hope that we have adequately addressed the reviewers' comments, and we look forward to receiving a favorable outcome on our paper.

Yours Sincerely,

Augustus Osborne

Corresponding Author
